# IgA nephropathy with mimicking Fabry disease: A case report and literature review

**DOI:** 10.1097/MD.0000000000031060

**Published:** 2022-10-21

**Authors:** Liping Sun, Xinyi Zi, Zhen Wang, Xinzhou Zhang

**Affiliations:** a Key Renal Laboratory of Shenzhen, Shenzhen, China; b Department of Nephrology, Shenzhen People’s Hospital (The Second Clinical Medical College, Jinan University, The First Affiliated Hospital, Southern University of Science and Technology), Shenzhen, China.

**Keywords:** diagnosis, Fabry disease, IgA nephropathy, renal biopsy, zebra body

## Abstract

Fabry disease (FD) is a rare, inherited disease lysosomal storage disorder caused by the lack of an alpha-galactosidase enzyme. This genetic disease can affect both men and women. The understanding of FD is very important as this condition can be effectively treated. For women who may exhibit normal residual enzyme activity, the diagnosis is more challenging.

**Case presentation:**

Herein, we reported on a case of IgA nephropathy and renal disease that mimicked FD in a female patient. The presence of zebra bodies in the cytoplasm of glomerular podocytes is widely accepted as a hallmark pathological manifestation of FD. In the present case, renal biopsy analysis revealed the presence of zebra bodies; however, genetic testing indicated that the patient did not have FD. The mechanisms and causes of zebra body formation remained unclear in the present case. However, the patient responded well to treatment with an angiotensin receptor blocker.

**Conclusions:**

The reported findings can be useful for the differential diagnosis of FD and renal diseases in the future. Our results also highlight the clinical significance of zebra bodies in renal disease.

## 1. Introduction

Fabry disease (FD) is an X chromosome hereditary disorder caused by a mutation of the alpha-galactosidase (α-GalA) gene^[1]^ that causes partial or complete deletion of the functions of α-GalA. Globotriaosylceramide (GL3) is metabolized by α-GalA, which accumulates in the lysosome of cells and leads to abnormal cell structure and function. FD is a multisystem lysosomal storage disease that affects the kidneys, cardiovascular system, skin, eyes, nervous system, and other tissues and organs. A large number of myelin-like bodies in the cytoplasm of glomerular podocytes are the characteristic pathological manifestation. Furthermore, a decrease in α-GalA activity and an abnormal increase in GL3 and its metabolite lyso-globotriaosylceramide (lyso-Gb3) are considered as clinical indicators for the diagnosis of FD. Nevertheless, 30% of women may present with normal α-GalA activity because the defective gene can be compensated by a functional copy of the same gene on another X chromosome. Consequently, this type of examination is not recommended for women, who can benefit more from a screening strategy that combines enzyme activity with lyso-GL3 levels, thus improving the accuracy of disease detection. Therefore, genetic testing is the gold standard for diagnosing FD.^[2–6]^ In addition, some drugs, such as hydroxychloroquine, chloroquine, and amiodarone, can decrease α-GalA activity and induce the deposition of phospholipid, making the renal histological characteristics similar to those of FD.^[7,8]^ Phospholipid deposition can induce the formation of anti-phospholipid autoantibodies,^[9]^ while 45% of patients with FD develop anti-phospholipid autoantibodies.^[10]^ The lysosomal deposition of Gb3 can interfere with some immunological processes such as cytokines release, antigen presentation to immune-competent cells.^[5]^ This indicates a simultaneous autoimmune response and may explain why FD mimics immune-associated glomerular diseases like IgA nephropathy. Mesangial proliferation lesions, accompanied by diffuse granular IgA deposits in the mesangial area, are consistent with the diagnosis of IgA nephropathy. At the same time, secondary IgA nephropathy such as rheumatoid arthritis, ankylosing spondylitis, allergic purpura nephritis, and chronic hepatitis B can be excluded.

As FD mimics the features of other renal diseases, its differential diagnosis is very important. The clinical diagnosis of FD is mainly based on detailed family history, abnormal enzyme and LysoGb3 levels, typical renal histopathological features, and genetic testing.^[11]^ Electron microscopy examination of renal biopsy samples and observed zebra bodies is important for diagnosing FD and eliminating other primary and secondary renal diseases.^[6,12]^ Angiotensin-converting enzyme inhibitor or angiotensin receptor blocker (ARB) is individually used for the nonspecific adjunctive treatment of FD. At present, specific enzyme replacement, enzyme enhancement, and gene therapy are the main treatment methods for this disease.^[13,14]^

In this study, we reported a rare case of a patient who exhibited histological signs of FD with IgA nephropathy, while genetic testing indicated that the patient did not have FD.

## 2. Case presentation

This study was approved by our hospital, and written informed consent was obtained from the patient for the publication of this case report.

A 30-year-old female patient was admitted to the hospital on January 15, 2020, with a history of proteinuria for more than 2 years that has aggravated over the previous 2 weeks. There was no obvious relationship between posture changes and exercise, and no distal paresthesia. She denied having a history of hypertension and diabetes or using drugs such as hydroxychloroquine, chloroquine, and amiodarone, which have previously been reported to induce zebra body formation. Neither of her parents had a history of kidney disease, while routine urine tests showed no abnormalities. Physical examination showed that her temperature was 36.5°C; her blood pressure, 120/73 mmHg; and her heart rate, 82 bpm. The patient did not have any rashes on the body, abnormalities related to the eyes and ears, or edema in her lower extremities on any of the sides.

The results of routine urine tests were as follows: urine protein, 1+~2+; red blood cells, 0~++/HP; 24-h urine protein, 1400 mg. Blood biochemistry findings showed that renal function was normal: serum creatinine level (SCr), 66 μmol/L; estimated glomerular filtration rate, 117 mL/min/1.73 m^[2]^; plasma albumin level, 44.6 g/L. The results of liver function, immunofixation electrophoresis, hepatitis virus, human immunodeficiency virus, syphilis, echocardiography, and ophthalmic examinations were negative. The results of immunology tests for immunoglobulins, complements, double-stranded DNA, antinuclear antibody, Sjögren’s syndrome A, Sjögren’s syndrome B, antineutrophil cytoplasmic antibody, antistreptolysin O, and rheumatoid factor were also normal. Abdominal ultrasound examination showed that both kidneys had normal size (left kidney: 108 × 46 mm, right kidney: 105 × 42 mm) and morphology. Further, no abnormalities were detected on the chest radiograph, electrocardiogram, cardiac color Doppler ultrasonogram, or craniocerebral magnetic resonance images.

**Figure 1.**
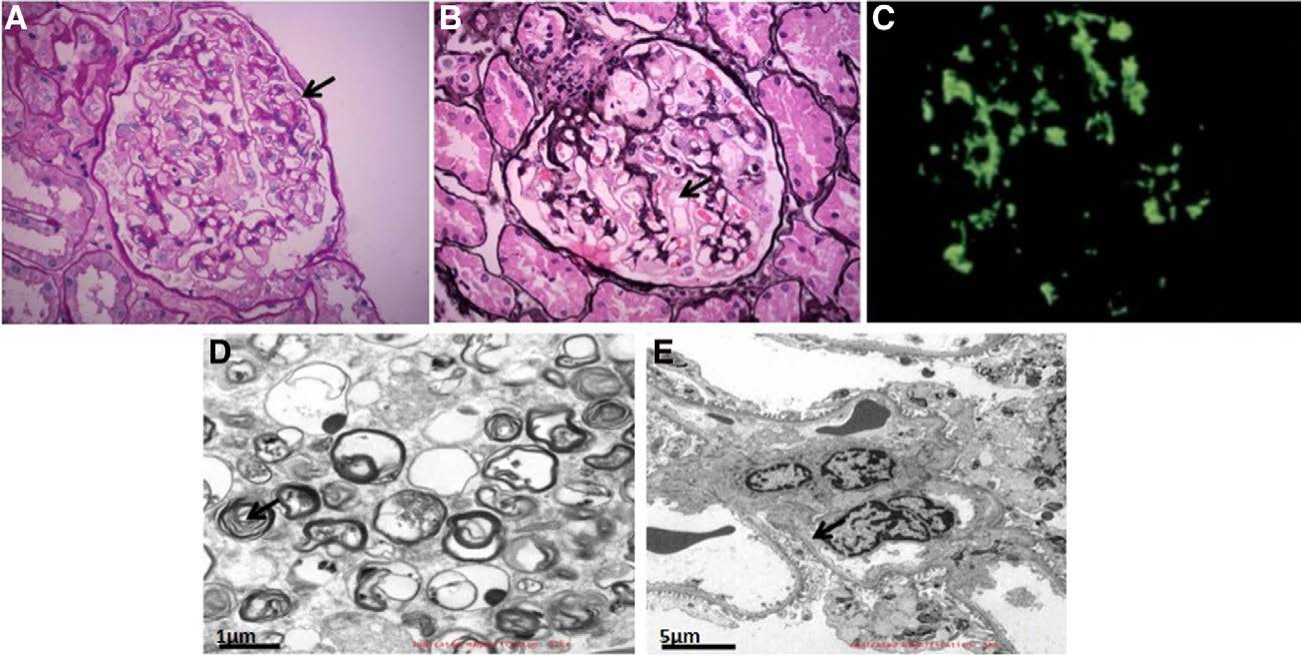
Morphological features of the renal biopsy tissue. (A) Hematoxylin-eosin stain was observed in the cytoplasm of glomerular podocytes (toluidine blue staining, ×400). (B) The glomerular podocytes were significantly swollen and exhibited foam-like changes (PASM, Periodic Acid-Silver Methenamine, ×400). (C) Immunofluorescence staining analysis (×400) demonstrated strongly positive staining for IgA. (D & E) Myeloid bodies and zebra bodies were formed in the podocytes (Arrow pointed), and a small amount of electron-dense deposits were observed in the mesangial area (electron microscope).

Pathological examination of the renal biopsy sample under a light microscope (Fig. 1A, B) showed that eight glomeruli were present, but they did not exhibit glomerular sclerosis or segmental glomerular sclerosis. A slight increase in the volume of the glomerular mesangial matrix, swelling of some podocytes, vacuolar degeneration of renal tubular epithelial cells, a small area of focal atrophy (5%), small focal inflammatory cell infiltration in the renal interstitium, and small-size arteries were observed. However, there was no obvious fibrosis or obvious disease in the tube wall. Immunofluorescence analysis of paraffin-embedded sections of renal biopsy tissue (Fig. 1C) did not demonstrate any glomeruli, but IgA (+ to 2+), IgM (+/−), IgG, C3, C1q, and Fib were observed. Still, the tissue was not positive for Alb, while small droplets of renal tubular reabsorption were Alb positive. Electron microscope specimens were stained with toluidine blue. There was no obvious proliferation of cells in the glomerular capillaries, the capillary loops were open, and parietal cells did not show proliferation. No obvious thickening of the basement membrane (thickness = 260–360 nm) was observed; however, swelling of epithelial cells, vacuolar degeneration (indicated by a foam-like appearance), increase in the number of secondary lysosomes, myeloid corpuscles (zebra bodies), and segmental fusion of the foot process were observed (Fig. 1D, E). No electron-dense deposits were observed in the subepithelial and basement membrane, but the slight proliferation of mesangial cells and the surrounding matrix and a small amount of electron-dense deposits in the mesangial area were observed. No medullary bodies were detected in renal tubular epithelial cells, arteriole endothelial cells, or smooth muscle cells. Based on these findings, the pathological diagnosis was IgA nephropathy (Oxford type M1E0S0T0C0) with renal FD. However, analysis of the patient’s serum sample for plasma lyso-Gb3 showed a concentration of <0.55 ng/mL (normal value, <1.11 ng/mL), and genetic testing revealed no abnormalities. These findings were contradictory to the diagnosis of FD. The patient’s condition was stable, and she was discharged on January 21, 2020. The final diagnosis was IgA nephropathy (Oxford classification M1E0S0T0C0) with a renal disease that mimicked FD. After the renal biopsy, the patient was administered losartan potassium, 100 mg, q.d. (an ARB, Hangzhou Moshadong Pharmaceutical Co., Ltd.), without any hormone or immunosuppressant.

After 1 year follow-up at our department, the patient insisted on taking losartan potassium 100 mg (q.d.) for about a year, and no other drugs were added to her regimen. The 24-h urinary protein concentration gradually decreased till almost reaching normal levels (March 2020: 1019 mg, April 2020: 856 mg, July 2020: 470 mg, October 2020: 360 mg, January 2021: 190 mg). The patient’s renal function remained stable for a long time (March 2020: SCr, 73 μmol/L, BUN, 4.6 mmol/L, July 2020: SCr, 68 μmol/L, BUN, 4. 1 mmol/L, October 2020: SCr, 64 μmol/L, BUN, 4. 7 mmol/L, January 2021: SCr, 71 μmol/L, BUN, 4. 2 mmol/L).

## 3. Discussion

Herein, we reported the case of a young female patient who exhibited a chronic renal disease with the characteristic renal pathological features of IgA nephropathy and renal FD. Consequently, the patient was pathologically diagnosed with FD complicated with IgA nephropathy. However, FD is unlikely in a female with normal levels of α-GalA activity and lyso-Gb3,^[15]^ and genetic testing showed that the patients did not have an α-GalA mutation, so FD was ruled out, and she was finally diagnosed with IgA nephropathy and renal disease that mimics FD.

FD with IgA nephropathy is an extremely rare occurrence. So far, the largest sample reported internationally included a series of 6 cases reported by Yang et al in China in 2017.^[10]^ Their literature analysis revealed that the clinical manifestations and renal pathological morphology of FD with IgA nephropathy were diverse in those patients who were proven to have FD. Based on their findings and our experience from the present case, it is recommended to combine family history, clinical manifestations, α-GalA activity, genetic testing, and pathological features of renal biopsy samples, especially electron microscopy observations, when making the diagnosis.

Our patient was a young woman who had proteinuria and hematuria for more than two years at the time of treatment. Immunofluorescence examination of renal tissue under an electron microscope showed IgA and electron-dense deposits in the mesangial area, and these features were consistent with the diagnosis of IgA nephropathy. Glomerular sclerosis, interstitial fibrosis, and podocyte vacuolization were not obvious under the light microscope. Electron microscopy revealed zebra bodies in one podocyte, which is a typical pathological feature of FD. However, the patient did not present with extrarenal manifestations such as skin keratinization, tumor blood vessels, corneal opacity, limb pain, or cardiovascular, nervous system lesions. The patient had no personal or family history supporting the diagnosis of FD, and the results of lyso-GL3 and the genetic tests were normal. Some studies have reported that renal phospholipidosis also occurs in patients taking several drugs,^[16]^ including amiodarone, chloroquine, aminoglycosides, chlorpromazine, fluoxetine, sertraline, and azithromycin. Nonetheless, our patient did not take any of these drugs.^[7,8,17]^ Additionally, although a relationship between phospholipid metabolism and antiphospholipid antibodies has been proposed in some case reports, the exact mechanism is still unclear. Therefore, based on these previous findings, this patient probably had a rare type of IgA nephropathy that was associated with zebra bodies but was not related to hydroxychloroquine use. Unfortunately, the causative mechanisms of zebra bodies are still unclear and need to be further studied.

Patients with renal involvement in FD often have a poor prognosis.^[18]^ Compared with FD, the prognosis of IgA nephropathy is relatively good. The basic treatment for IgA nephropathy consists of removing risk factors, in particular hypertension, with blockade of the renin-angiotensin-aldosterone system. In this case, the patient was only treated with ARB, and after one year, the patient’s 24-h urinary protein significantly decreased and remained at a low level, and her renal function remained stable. These results suggested that this treatment plan was effective, even though FD was ruled out.

To conclude, this was a rare case of a patient who had subtle histologic renal FD features co-existing with IgA nephropathy, where noninvasive biomarkers and genetic testing contradicted the diagnosis of FD. These findings might be useful for the differential diagnosis of FD and renal diseases in the future. They also may further the research on the clinical significance of zebra bodies and kidney phospholipid disease.

## Author contributions

All authors contributed to the study’s conception and design. Material preparation, data collection, and analysis were performed by Liping Sun and Xinzhou Zhang. The first draft of the manuscript was written by Liping Sun, and all authors commented on previous versions of the manuscript. All authors read and approved the final manuscript.

**Conceptualization:** Liping Sun, Xinyi Zi, Zhen Wang, Xinzhou Zhang.

**Formal analysis:** Liping Sun, Xinzhou Zhang.

**Investigation:** Liping Sun, Xinzhou Zhang.

**Methodology:** Liping Sun, Xinzhou Zhang.

**Writing – original draft:** Liping Sun.

**Writing – review & editing:** Liping Sun, Xinyi Zi, Zhen Wang, Xinzhou Zhang.
